# Magnetic Properties of A_2_Ni_2_TeO_6_ (A = K, Li): Zigzag Order in the Honeycomb Layers of Ni^2+^ Ions Induced by First and Third Nearest-Neighbor Spin Exchanges

**DOI:** 10.3390/ma15072563

**Published:** 2022-03-31

**Authors:** Tatyana Vasilchikova, Alexander Vasiliev, Maria Evstigneeva, Vladimir Nalbandyan, Ji-Sun Lee, Hyun-Joo Koo, Myung-Hwan Whangbo

**Affiliations:** 1 Department of Low Temperature Physics and Superconductivity, Lomonosov Moscow State University, Moscow 119991, Russia; t_vasilchikova@mig.phys.msu.ru; 2 Quantum Functional Materials Laboratory, National University of Science and Technology “MISiS”, Moscow 119049, Russia; 3 Department of Theoretical Physics and Applied Mathematics, Ural Federal University, Ekaterinburg 620002, Russia; 4 Faculty of Chemistry, Southern Federal University, Rostov-on-Don 344090, Russia; maevstigneeva@gmail.com (M.E.); vbn@sfedu.ru (V.N.); 5 Department of Chemistry and Research Institute for Basic Sciences, Kyung Hee University, Seoul 02447, Korea; ljisun200@khu.ac.kr (J.-S.L.); whangbo@ncsu.edu (M.-H.W.); 6 Department of Chemistry, North Carolina State University, Raleigh, NC 27695, USA

**Keywords:** metaloxides, honeycomb lattice, long-range order, short-range order, first principles calculations

## Abstract

The static and dynamic magnetic properties and the specific heat of K_2_Ni_2_TeO_6_ and Li_2_Ni_2_TeO_6_ were examined and it was found that they undergo a long-range ordering at *T_N_* = 22.8 and 24.4 K, respectively, but exhibit a strong short-range order. At high temperature, the magnetic susceptibilities of K_2_Ni_2_TeO_6_ and Li_2_Ni_2_TeO_6_ are described by a Curie–Weiss law, with Curie-Weiss temperatures Θ of approximately −13 and −20 K, respectively, leading to the effective magnetic moment of about 4.46 ± 0.01 *μ*_B_ per formula unit, as expected for Ni^2+^ (*S* = 1) ions. In the paramagnetic region, the ESR spectra of K_2_Ni_2_TeO_6_ and Li_2_Ni_2_TeO_6_ show a single Lorentzian-shaped line characterized by the isotropic effective *g*-factor, *g* = 2.19 ± 0.01. The energy-mapping analysis shows that the honeycomb layers of A_2_Ni_2_TeO_6_ (A = K, Li) and Li_3_Ni_2_SbO_6_ adopt a zigzag order, in which zigzag ferromagnetic chains are antiferromagnetically coupled, because the third nearest-neighbor spin exchanges are strongly antiferromagnetic while the first nearest-neighbor spin exchanges are strongly ferromagnetic, and that adjacent zigzag-ordered honeycomb layers prefer to be ferromagnetically coupled. The short-range order of the zigzag-ordered honeycomb lattices of K_2_Ni_2_TeO_6_ and Li_2_Ni_2_TeO_6_ is equivalent to that of an antiferromagnetic uniform chain, and is related to the short-range order of the ferromagnetic chains along the direction perpendicular to the chains.

## 1. Introduction

**Figure 1:**
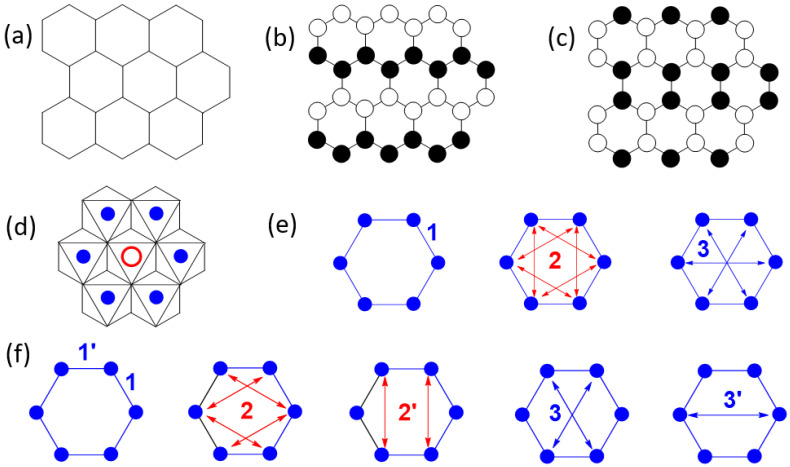
(**a**) A simplified view of a honeycomb layer made up of magnetic ions. (**b**) A zigzag order of magnetic moments in a honeycomb layer. (**c**) A stripy order of magnetic moments in a honeycomb layer. (**d**) A honeycomb layer of magnetic ions (solid blue circles) in which the center of every hexagon of magnetic ions is occupied by a cation (e.g., Na^+^ in Na_2_IrO_3_, Sb^5+^ in A_3_Ni_2_SbO_6_, Te^6+^ in A_2_Ni_2_TeO_6_, (P-P)^8+^ dimer in NiPS_3_). (**e**) First NN spin exchange (*J*_1_), second NN spin exchange (*J*_2_), and third NN spin exchange (*J*_3_) in a honeycomb layer made up of regular hexagons of magnetic ions. (**f**) First NN spin exchange (*J*_1_ and *J*_1_′), second NN spin exchange (*J*_2_ and *J*_2_′), and third NN spin exchange (*J*_3_ and *J*_3_′) in a honeycomb layer made up of slightly distorted hexagons of magnetic ions (e.g., 2-fold rotational symmetry).

Compounds with honeycomb layers of magnetic ions attracted much attention in the field of low-dimensional magnetism [1], owing in part to Kitaev’s conjecture [2] that they can have gapped and gapless liquid-like ground states in a certain range of spin exchange parameters. It has been elusive to find honeycomb-layered (Figure 1a) magnetic systems confirming Kitaev’s conjecture because of the inevitable interlayer interactions, which can lead to a three-dimensional (3D) long-range ordering. In real honeycomb-layered materials, anisotropic in-plane Kitaev interactions compete with isotropic Heisenberg interactions. The ordering of magnetic moments in honeycomb layers can be zigzag (Figure 1b) or stripy (Figure 1c) [3]. Kitaev’s conjecture was originally analyzed for the spin *S* = 1/2 case, but it has subsequently been extended to *S* > 1/2 systems (in particular, *S* = 1), although these systems are not exactly solvable [4]. It was shown that the bond-dependent Kitaev interaction model can be realized in two-dimensional (2D) Mott insulators if there exists strong Hund coupling of electrons at the cation sites and strong spin-orbit coupling at the anion sites [4]. The iridate Na_2_IrO_3_ with honeycomb layers of low-spin Ir^4+^ (*d*^5^, *S* = 1/2) ions [5,6,7] and the ruthenium chloride α-RuCl_3_ with honeycomb layers of low-spin Ru^3+^ (*d*^5^, *S* = 1/2) ions [8,9,10] were examined as the *S* = 1/2 systems that can capture the basics of Kitaev magnetism. The iridate β-Li_2_IrO_3_ has also been examined for Kitaev magnetism, but its Ir^4+^ ions form a 3D framework rather than honeycomb layers [11,12]. The magnetic structure in the honeycomb layers of Na_2_IrO_3_ is consistent with a zigzag order (i.e., FM zigzag chains of Ir^4+^ ions are antiferromagnetically coupled, Figure 1b) or a stripy order (i.e., AFM zigzag chains of Ir^4+^ ions are ferromagnetically coupled, Figure 1c) [6]. The magnetic structure of α-RuCl_3_ is more consistent with a zigzag order than with a stripy order [9]. The layered-phase A_3_Ni_2_SbO_6_ (A = Li, Na) [13,14] consists of honeycomb layers of *S* = 1 ions (i.e., Ni^2+^ ions). Each honeycomb layer consists of edge-sharing NiO_6_ octahedra each containing a Ni^2+^ ion, in which every hexagon of Ni^2+^ ions has its center occupied by a Sb^5+^ cation to form a SbO_6_ octahedron (Figure 1d). The magnetic structure of A_3_Ni_2_SbO_6_ (A = Li, Na) in the honeycomb layers of Ni^2+^ ions is described by a zigzag order [13], similar to the one found for the *S* = 1/2 systems Na_2_IrO_3_ and α-RuCl_3_. 

So far, it is not clear why the honeycomb layers of these magnetic systems form a zigzag order, namely, why they adopt FM zigzag chains that are antiferromagnetically coupled. To understand the cause for this ordering, it is necessary to know the spin exchanges between the first, second, and third nearest-neighbor (NN) magnetic ions, which are *J*_1_, *J*_2_, and *J*_3_ depicted in Figure 1e, respectively, when a honeycomb layer is made up of regular hexagons of magnetic ions. However, when these hexagons are lower in symmetry (e.g., 2-fold rotational symmetry), the first, second, and third NN spin exchanges are each split into two different ones, as depicted in Figure 1f. Zvereva et al. evaluated three spin exchanges of A_3_Ni_2_SbO_6_ (A = Li, Na) [13] (namely, the first NN exchanges *J*_1_ and *J*_1_′ as well as the third NN exchanges *J*_3_ and *J*_3_′, Figure 1f), and found that *J*_1_ is AFM, *J*_1_′ is FM, while the third NN spin exchanges *J*_3_ and *J*_3_′ are practically zero. Although this is consistent with a zigzag order in A_3_Ni_2_SbO_6_ (A = Li, Na), we note that a zigzag order also occurs in the honeycomb layers of M^2+^ (M = Fe, Co, Ni) ions in the sulfides MPS_3_, in which each hexagon of M^2+^ ions has its center occupied by a P_2_S_6_^4−^ ion (Figure 1d) to form MS_6_ octahedra. The strongest spin exchange of NiPS_3_ was found to be the third NN exchanges (*J*_3_ and *J*_3_′), which are strongly AFM, while the first NN spin exchanges (*J*_1_ and *J*_1_′) are FM and are weaker than the third NN exchanges by a factor greater than three [15]. Thus, the zigzag order of NiPS_3_ is caused by the strongly AFM third NN spin exchanges. This finding makes it necessary to check if the third NN spin exchanges vanish in A_3_Ni_2_SbO_6_ (A = Li, Na) as reported [13].

Honeycomb layers of Ni^2+^ ions similar to those of A_3_Ni_2_SbO_6_ (A = Li, Na) are also present in A_2_Ni_2_TeO_6_ (A = K, Li) [16,17,18,19]. Formally, A_2_Ni_2_TeO_6_ results from A_3_Ni_2_SbO_6_ by replacing the Sb^5+^ ion with a Te^6+^ ion, by removing one cation A^+^ to satisfy the charge balance requirement, and by changing the layer stacking mode leading to trigonal prismatic and tetrahedral coordination for K^+^ and Li^+^, respectively. In the present work, we probed the static and dynamic magnetic properties of A_2_Ni_2_TeO_6_ (A = K, Li) and found that they are very similar to those of A_3_Ni_2_SbO_6_ (A = Li, Na), and we determined the spin exchanges (Figure 1f) of A_2_Ni_2_TeO_6_ using the energy-mapping analysis [20,21] to show that the honeycomb layers of not only A_2_Ni_2_TeO_6_ but also A_3_Ni_2_SbO_6_ have a zigzag magnetic order for the same reason as found for NiPS_3_. Thus, the cause for the zigzag order of not only A_2_Ni_2_TeO_6_ (A = K, Li) but also A_3_Ni_2_SbO_6_ (A = Li, Na) is the third NN spin exchange, which is strongly AFM, as found for NPS_3_. 

Both static and dynamic magnetic properties of A_2_Ni_2_TeO_6_ (A = K, Li) evidence the behavior inherent in a magnet with reduced dimensionality, and the long-range order in these compounds is preceded by a short-range order. These observations are fully supported by specific heat measurements, which reveal sharp λ-type singularities establishing the occurrence of a Néel order. We show that the honeycomb layers of A_2_Ni_2_TeO_6_ (A = K, Li) and Li_3_Ni_2_SbO_6_ adopt a zigzag order, in which zigzag ferromagnetic chains are antiferromagnetically coupled, and that the short-range order of Li_2_Ni_2_TeO_6_ and K_2_Ni_2_TeO_6_ arises from the short-range order of the ferromagnetic chains along the direction perpendicular to the chains.

## 2. Sample Preparation and X-ray Diffraction

### 2.1. K_2_Ni_2_TeO_6_

Light-green samples of K_2_Ni_2_TeO_6_ were prepared by conventional solid-state synthesis. Since the material is highly hygroscopic, it was necessary to protect it from the atmospheric moisture (see the Supplementary Materials for details). In agreement with the previous data [18], the XRD pattern (Supplementary Figure S1) shows that our sample of K_2_Ni_2_TeO_6_ represents a hexagonal superlattice of the P2 type, space group *P6_3_*/*mcm*, with only weak extra reflections. Least squares refinements resulted in the lattice parameters *a* = 5.258(3), *c* = 12.417(1) Å, and *c/a* = 2.362, which are consistent with the literature data [18]. The small differences in the absolute values are systematic and may hence be due to uncertainties in our refined sample displacement and/or in the wavelengths used. Thus, it is important to compare the axial ratios. Masese et al. [18] reported a slightly higher *c/a* ratio of 2.370, together with the dark green color of their sample, which suggests the presence of some Ni^3+^ ions, presumably due to potassium deficiency. Chemical analysis of our light-green sample by reverse redox titration [22] yielded the oxidation state of 2.01 ± 0.01 for Ni, thus confirming the stoichiometry. In contrast, our potassium-deficient samples were black, containing a considerable amount of Ni^3+^ ions and exhibiting much larger *c/a* ratios. This is a common feature of the present class of structures: the oxidation of an octahedral cation results in the *a*-axis contraction, and the loss of the interlayer alkali ions, in the *c*-axis expansion. However, the deviation from stoichiometry in the reported structure [18] is evidently small and the reported structure is quite reasonable, so we did not attempt a re-refinement.

### 2.2. Li_2_Ni_2_TeO_6_

We prepared Li_2_Ni_2_TeO_6_ from Na_2_Ni_2_TeO_6_ by an ion-exchange reaction [16] (see the Supplementary Materials for details). Previously, the XRD powder pattern (Supplementary Figure S2) was indexed in the space group *Cmca* by analogy with T2-Li_2_NiMn_2_O_6_ [23]. The same preparation route and the same space group were adopted by Grundish et al. [17], and the orthorhombic lattice parameters are in reasonable agreement (Table 1). Li_2_Ni_2_TeO_6_ is not isostructural with its sodium precursor (*P6_3_/mcm*) because small Li^+^ ions cannot be accommodated in the trigonal prismatic sites (i.e., Na^+^ ion sites), so the adjacent honeycomb (Ni_2_TeO_6_)^2−^ layers are shifted to provide tetrahedral interlayer sites suitable for Li^+^ ions, as found in T2-Li_2_NiMn_2_O_6_ [23]. Unfortunately, crystal structure refinement of the T2-type Li_2_Ni_2_TeO_6_ [17] resulted in unrealistic bond lengths and bond valence sums (BVSs) (Table 2), and our refinement results were not better. The reasons for this might be: (i) Admixture of foreign phase(s): the zoomed-in view of the XRD pattern (Figure 5 in [17]) reveals a strong unindexed reflection at 2Θ ≈ 17.53°. Visually, it might seem to be the α2 component of the strongest reflection (2Θ ≈ 17.38°), but their separation is three times larger than the doublet separation. Much weaker shoulders are seen in our patterns as well (Figure S2). (ii) The powder patterns are diffuse in nature arising from the stacking faults due to the layer gliding, which is induced by the ion-exchange transforming the space group of the crystal structure from *P6_3_/mcm* to *Cmca*. (iii) X-ray diffraction measurements have a low sensitivity to the positions of light atoms (Li and O) in the presence of heavy elements (Te and Ni). Thus, in the X-ray crystal structure reported for Li_2_Ni_2_TeO_6_ [17], some Li, O1, and O2 positions are known only in three decimal places. Thus, it is important to have more accurate atomic positions. The crystal structure of Li_2_Ni_2_TeO_6_ was optimized by DFT calculations [17], but the resulting atomic positions were not reported. Thus, we optimized the crystal structure of Li_2_Ni_2_TeO_6_ by DFT + U calculations with U_eff_ = 4 eV, and the optimized atomic positions are summarized in Supplementary Table S1. 

The average Li-O, Ni-O, and Te-O bond lengths of Li_2_Ni_2_TeO_6_, determined from the X-ray diffraction and our DFT + U optimized structures, are compared in Table 2, and so are the oxidations states for the Li, Ni, Te, and O atoms of Li_2_Ni_2_TeO_6_ obtained from bond valence sum (BVS) calculations based on the two crystal structures. According to the optimized crystal structure, the oxidation states of Li and Ni are very close to those expected from the ionic electron counting scheme (+1 and +2, respectively). In contrast, the oxidation state of Te is considerably smaller than expected from the ionic electron counting scheme (i.e., +5.29 vs. +6), while that of O is considerably higher than expected (i.e., −1.72/−1.87 vs. −2). 

## 3. Results and Discussion

### 3.1. Magnetic Properties

#### 3.1.1. Magnetic Susceptibility and Magnetization

**Figure 2:**
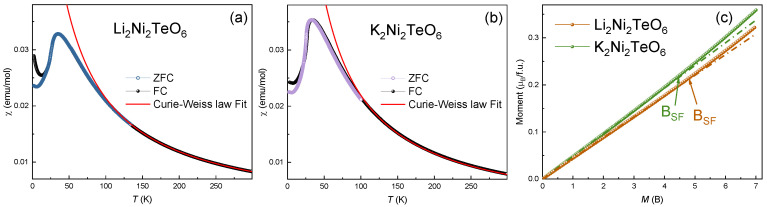
ZFC and FC magnetic susceptibilities of (**a**) Li_2_Ni_2_TeO_6_ and (**b**) K_2_Ni_2_TeO_6_ taken at *B* = 0.1 T. The solid lines are the fits by the Curie–Weiss law, dashed green lines—Rushbrook–Wood model fit. (**c**) Magnetizations of Li_2_Ni_2_TeO_6_ and K_2_Ni_2_TeO_6_ at *T* = 2 K. The fields of the spin–flop transition, B_sf_, are marked by arrows.

The magnetic susceptibilities, *χ*(T), of Li_2_Ni_2_TeO_6_ and K_2_Ni_2_TeO_6_ were measured at *B* = 0.1 T in the temperature range of 2–300 K using a Quantum Design PPMS-9T system, and their isothermal magnetizations, *M*(*B*), using a Quantum Design MPMS-7T SQUID-VSM magnetometer under an external field up to 7 T at various temperatures after cooling the sample at zero magnetic field. Results of zero-field-cooled (ZFC) and field-cooled (FC) measurements carried out for Li_2_Ni_2_TeO_6_ and K_2_Ni_2_TeO_6_ as well as magnetization curves are shown in Figure 2a–c. A small divergence between the results of ZFC and FC measurements indicates the presence of a modest spin disorder, due most likely to impurity-related effects. On lowering the temperature, the magnetic susceptibilities of both Li_2_Ni_2_TeO_6_ and K_2_Ni_2_TeO_6_ exhibit a broad maximum, *χ*_max_, at *T*_max_ ≈ 34 K, then the value drops by about one third of *χ*_max_. The values of the Néel temperatures, *T_N_*, deduced as the temperature, *T,* at which the derivative d*χ*(*T*)/d*T* shows a maximum (not shown), are ~22.8 K for K_2_Ni_2_TeO_6_ and ~24.4 K for Li_2_Ni_2_TeO_6_. These *T_N_* values are considerably lower than *T*_max_, signaling the presence of strong short-range correlations. We note that AFM uniform chains exhibit a broad magnetic susceptibility maximum due to their short-range magnetic order. The occurrence of a magnetic susceptibility maximum in A_2_Ni_2_TeO_6_ (A = Li, K) and A_3_Ni_2_SbO_6_ (A = Li, Na) [13,14] suggests that their magnetic properties possess a one-dimensional (1D) character, although their honeycomb magnetic lattices are 2D in nature.

The high-temperature magnetic susceptibility can be fitted by the Curie–Weiss law plus a temperature-independent term, *χ*_0_:

(1) $$\chi =\chi_{0}+\frac{C}{T-\Theta }$$

where Θ is the Weiss temperature, and *C* is the Curie constant, *C* = *N*_A_*μ*_eff_^2^*μ*_B_^2^/3*k*_B_ (*μ*_eff_ is the effective magnetic moment, while *N*_A_, *μ*_B_, and *k*_B_ are Avogadro’s number, Bohr magneton, and Boltzmann constant, respectively). The diamagnetic contributions of K_2_Ni_2_TeO_6_ and Li_2_Ni_2_TeO_6_ were estimated to be *χ*_0_ = −1.38 10^−4^ and −1.1 10^−4^ emu/mol, respectively, by summing the Pascal’s constants [26]. The *χ*_0_ values were fixed to reduce the number of variable parameters during the fitting analysis. From this analysis, it was found that Θ = −13 K for K_2_Ni_2_TeO_6_ and −20 K for Li_2_Ni_2_TeO_6_, implying the presence of dominant antiferromagnetic interactions while, per formula unit (f.u.), *μ*_eff_ = 4.45 *μ*_B_ for K_2_Ni_2_TeO_6_ and 4.47*μ*_B_ for Li_2_Ni_2_TeO_6_. The effective *g*-factor obtained from ESR data (see below) is about *g* ≈ 2.2. The result hence well agrees with the theoretical estimate of the effective magnetic moment equal to 4.4 *μ*_B_/f.u. for both compounds, where *n* is the number of Ni^2+^ ions per formula unit, assuming Ni^2+^ in a high-spin configuration (*S* = 1).

(2) $$\mu_{theor}=\sqrt{g^{2}nS\left(S+1\right)\mu_{B}^{2}}$$

The magnetic susceptibility of A_2_Ni_2_TeO_6_ (A = K, Li) can be analyzed on the basis of the high-temperature series expansion (HTSE) approach for a 2D planar honeycomb lattice using the Rushbrook and Wood model [27]. Then, the *χ*(*T*) curve in the paramagnetic region can be described by:

(3) $$\chi =\frac{2Ng^{2}\beta^{2}}{3kT}\cdot \frac{1}{1+Ax+Bx^{2}+Cx^{3}+Dx^{4}+Ex^{5}+Fx^{6}}$$

where *x* = |*J|*/*kT*, *A* = 4, *B* = 7.333, *C* = 7.111, *D* = –5.703, *E* = –22.281, and *F =* 51.737.^27^ Fitting the *χ*(*T*) curve by Equation (3) in the range of 50–300 K yields *J* = −8 ± 1 K for both compounds. The magnetization isotherms, *M*(*B*), taken at 2 K (Figure 2c) demonstrate upward deviations from the linear dependences, suggesting spin–flop transitions at *B*_SF_ ≈ 4.7 T and 4.4 T for Li_2_Ni_2_TeO_6_ and K_2_Ni_2_TeO_6_, respectively. Table 3 summarizes the parameters describing the magnetic subsystems of A_2_Ni_2_TeO_6_ (A = Li, K), obtained from the magnetic susceptibility and magnetization measurements.

#### 3.1.2. Electron Spin Resonance

**Figure 3:**
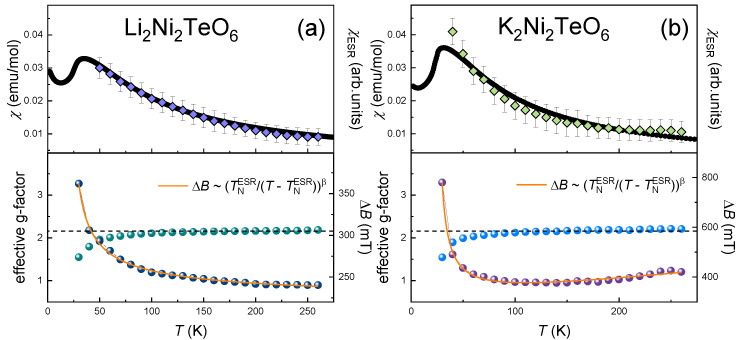
The temperature dependence of the main ESR parameters of (**a**) K_2_Ni_2_TeO_6_ and (**b**) Li_2_Ni_2_TeO_6_ derived from fitting the absorption line with the Lorentzian profile: the integral ESR intensity is shown in the upper panel, and the effective *g*-factor and the ESR linewidth, Δ*B*, in the lower panel. The orange solid curves represent an approximation in accordance with a modified Huber theory (Equation (5)), as described in the text for (**a**), and in the framework of Kawasaki–Mori–Huber theory (Equation (6)) for (**b**).

Electron spin resonance (ESR) studies were carried out using an X-band ESR spectrometer CMS 8400 (ADANI) (*f* ≈ 9.4 GHz, *B* ≤ 0.7 T), equipped with a low-temperature mount, operating in the range of *T* = 6–300 K. The effective *g*-factors were calculated using an external reference for the resonance field, i.e., BDPA (*a,g-*bisdiphenylene-*b*-phenylallyl), for which *g*_et_ = 2.00359. The ESR data in the paramagnetic phase (*T* > *T_N_*) show a single broad Lorentzian line-shape ascribable to Ni^2+^ ions in octahedral coordination [28] for both K_2_Ni_2_TeO_6_ and Li_2_Ni_2_TeO_6_ (Supplementary Figure S10). The main ESR parameters (effective *g*-factor, the ESR linewidth, and the integral ESR intensity) were deduced by fitting the experimental spectra with the Lorentzian profile [29]:

(4) $$\frac{dP}{dB}\propto \frac{d}{dB}\left[\frac{\Delta B}{\Delta B^{2}+{\left(B-B_{r}\right)}^{2}}+\frac{\Delta B}{\Delta B^{2}+{\left(B+B_{r}\right)}_{2}}\right]$$

where *P* is the power absorbed in the ESR experiment, *B_r_* is the resonance field, and Δ*B* is the linewidth. The integral ESR intensity, *χ*_ESR_, which is proportional to the number of magnetic spins, was estimated by double integration of the first derivative ESR spectrum, d*P*/d*B*. Evidently, the temperature dependence of *χ*_ESR_(*T*) follows the Curie–Weiss relationship and agrees well with the static magnetic susceptibility *χ*(*T*), as shown in the upper panels of Figure 3.

The average effective *g*-factor of 2.20 ± 0.03 remains almost temperature-independent in the paramagnetic phase down to ~100 K (lower panels of Figure 3), and then the visible shift of the resonant field to higher magnetic fields begins upon approaching the Néel temperature from above. This behavior implies the presence of strong short-range correlations at temperatures noticeably higher than *T_N_*, which is characteristic of the systems with spin frustration and low dimensionality [29].

The linewidth, Δ*B,* of K_2_Ni_2_TeO_6_ shows three different dynamic regimes: It decreases weakly and almost linearly on lowering the temperature down to ~150 K, then remains constant down to ~100 K. Upon a further decrease in the temperature, the absorption line broadens significantly and the ESR signal vanishes in the vicinity of the Néel temperature, indicating the opening of an energy gap for resonance excitations, e.g., due to the occurrence of a long-range order. Similar spin dynamics were observed recently for A_3_Ni_2_SbO_6_ (A = Li, Na) with a honeycomb lattice of Ni^2+^ ions [13]. Following the same procedure, we treated Δ*B*(*T*) in the frame of the critical broadening model using the modified Huber’s formula [30,31,32,33] with the third linear term to account for the Δ*B*(*T*) behavior over the whole temperature range:

(5) $$\Delta B\left(T\right)=\Delta B^{*}+A{\left[\frac{T_{N}^{\mathrm{ESR}}}{T-T_{N}^{\mathrm{ESR}}}\right]}^{\beta }+DT$$

where the first term ΔB^*^ describes the exchange narrowed linewidth, which is temperature-independent. The second term describes the critical behavior, with *T_N_*^ESR^ as the temperature of the order–disorder transition and *β* as the critical exponent. The third term relates to the temperature-linear spin-lattice relaxation term. The solid line on the lower panel of Figure 3a represents a least-squares-fitting of Δ*B*(*T*). The best fitting was obtained with ΔB^*^ = 290 ± 5 mT, *β* ≈ 1 ± 0.05, and *D* = 0.4 mT/K. Clearly, $T_{N}^{\mathrm{ESR}}$ is in good agreement with *T_N_*. According to Kawasaki’s approach [25,26], the absolute value of the critical exponent can be expressed as *β* = [(7 + *η*)*ν*/2 − 2(1 − ζ)], where *ν* describes the divergence of the spin-correlation length, *η* is a critical exponent for the divergence of static correlations, and ζ reflects the divergence of the specific heat. Using the values *η* = ζ = 0 and *ν* = 2/3 for 3D antiferromagnets in the Heisenberg model, *β* becomes 1/3. Thus, the value of *β* ≈ 1 extracted for K_2_Ni_2_TeO_6_ is noticeably higher than 1/3 but is still below the value expected for pure 2D antiferromagnets (i.e., *β* ≈ 3/2) [34,35], but it is quite comparable to the *β* reported for other related quasi-2D Ni^2+^ compounds A_3_Ni_2_SbO_6_ (A = Li, Na) [13] and Li_4_NiTeO_6_ [36]. According to Kawasaki–Mori–Huber theory, the temperature variation of Δ*B* of Li_2_Ni_2_TeO_6_ can be described as:

(6) $$\Delta B\left(T\right)=\Delta B^{*}+A{\left[\frac{T_{N}^{\mathrm{ESR}}}{T-T_{N}^{\mathrm{ESR}}}\right]}^{\beta }$$

The best agreement by the least square method was obtained with the following parameters: ∆B* = 217 ± 5 mT and *β* = 0.60 ± 0.05. Thus, the analysis of spin dynamics supports the picture of rather a 2D character of magnetic correlations for both K_2_Ni_2_TeO_6_ and Li_2_Ni_2_TeO_6_. Spin-dynamic parameters of the studied compounds are summarized in Table 4.

#### 3.1.3. Specific Heat

**Figure 4:**
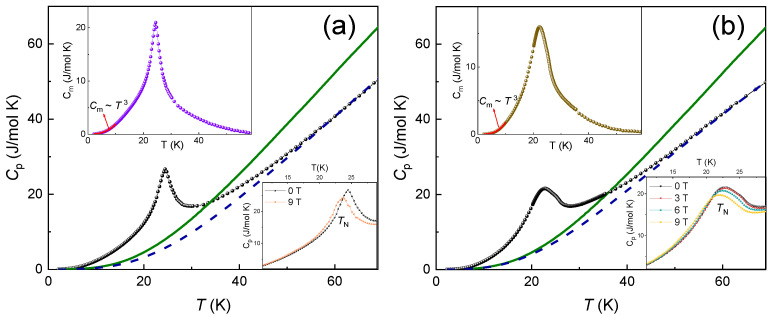
Temperature dependences of the specific heat in Li_2_Ni_2_TeO_6_ (**a**) and K_2_Ni_2_TeO_6_ (**b**). The data for Na_2_Zn_2_TeO_6_ are shown by solid lines. The dashed lines represent the reference curves obtained through the scaling procedure [39]. Lower insets represent *C*_p_(*T*) curves measured at various magnetic fields. Upper insets represent temperature dependences of magnetic specific heat, *C_m_*.

The specific heat, *C*_p_(*T*), of A_2_Ni_2_TeO_6_ (A = Li, K) has been measured using a relaxation method of a Quantum Design PPMS-9T. The data were collected at the zero magnetic field as well as under applied fields of 3, 6, and 9 T in the temperature range of 2–70 K. The *C*_p_(*T*) vs. *T* plots for K_2_Ni_2_TeO_6_ and Li_2_Ni_2_TeO_6_ are shown in Figure 4a,b. A λ-type peak is observed at *T_N_* for both K_2_Ni_2_TeO_6_ and A_2_Ni_2_TeO_6_, which clearly shows an occurrence of long-range antiferromagnetic order. These ordering temperatures, *T_N_*, coincide with the temperatures at which the peaks of the d(*χT*)/d*T* vs. *T* plot occur (i.e., the Fisher specific heat) [37,38], which is characteristic of low-dimensional antiferromagnets with strong short-range correlations. The positions of *T_N_* slightly shift toward the lower temperatures in the applied external magnetic field, as shown in the lower insets in Figure 4a,b, which is typical for antiferromagnetic compounds. To examine the magnetic contribution to specific heat in the title compounds, the *C_p_*(*T*) curve has been measured for the isostructural non-magnetic system Na_2_Zn_2_TeO_6_. Application of the scaling procedure [39] allows extracting magnetic specific heat, *C_m_*(*T*), as shown in the upper insets in Figure 4a,b. According to these data, the magnetic entropy, *S_m_*, released below *T_N_* is 6.74 and 5.71 J/mol·K for Li- and K-compounds, respectively. These values are to be compared with the thermodynamic limit *S_m_* = *nR*ln(2*S* + 1) = 18.27 J/mol·K at *n* = 2 and *S* = 1, meaning that the dominant part of the magnetic entropy is released above *T_N_*.

### 3.2. Spin Exchanges Leading to a Zigzag Magnetic Order

#### 3.2.1. Computational Details

To extract the values of the spin exchanges in A_2_Ni_2_TeO_6_ (A = K, Li) and Li_3_Ni_2_SbO_6_ (A = Li, Na), we carried out spin-polarized DFT calculations by using the frozen-core projector augmented plane wave met [40,41], encoded in the Vienna ab initio simulation package [42], and the PBE exchange-correlation functionals [43]. The electron correlation associated with the 3*d* states of Ni was taken into consideration by performing the DFT + U calculations [44] with the effective on-site repulsion U^eff^ = U − *J* on magnetic ions. All our DFT calculations used the plane wave cutoff energy of 450 eV and the threshold of 10^−6^ eV for self-consistent-field energy convergence. To relax the atom positions, DFT + U calculations (with U_eff_ = 4 eV) were performed using a set of (4 × 6 × 4) k-points with the criterion of 5 × 10^−4^ eV/Å for the relaxation of the atom positions. Our DFT + U calculations employed a set of (4 × 4 × 4) k-points for Li_2_Ni_2_TeO_6_, (5 × 5 × 3) k-points for K_2_Ni_2_TeO_6_, and (4 × 2 × 4) k-points for Li_3_Ni_2_SbO_12_. As a representative example for the A_3_Ni_2_SbO_6_ (A = Li, Na) family, we examined Li_3_Ni_2_SbO_12_ because Li_3_Ni_2_SbO_12_ is isostructural and isoelectronic with Na_3_Ni_2_SbO_12_, and because A_3_Ni_2_SbO_6_ (A = Li, Na) has already been studied [13]. In our DFT + U calculations, we employed the U_eff_ values of 3 and 4 eV, which lead to similar trends (see below).

#### 3.2.2. Spin Exchanges and Zigzag Order

**Figure 5:**
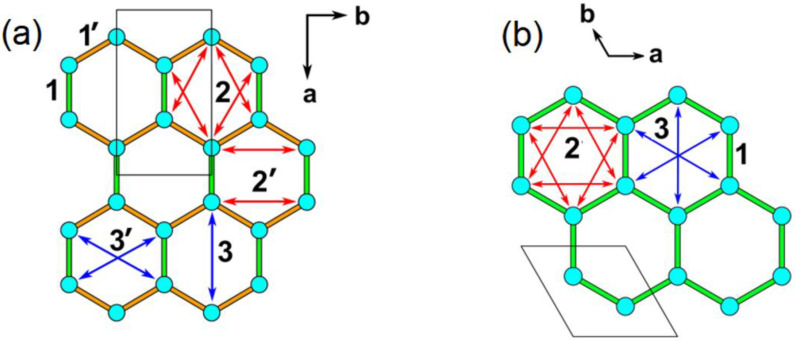
Intralayer spin exchanges defined for Li_2_Ni_2_TeO_6_ and Li_3_Ni_2_SbO_6_ in (**a**), and for K_2_Ni_2_TeO_6_ in (**b**).

The first, second, and third NN intralayer spin exchanges to consider for Li_2_Ni_2_TeO_6_ and Li_3_Ni_2_SbO_6_ are presented in Figure 5a, and those for K_2_Ni_2_TeO_6_ in Figure 5b. We evaluate these spin exchanges by using the energy-mapping analysis based on DFT calculations [15,20,21]. The three spin exchanges of K_2_Ni_2_TeO_6_ (Figure 5b) were determined using the four ordered spin states of Supplementary Figure S3. Similarly, the six intralayer spin exchanges of Li_2_Ni_2_TeO_6_ and Li_3_Ni_2_SbO_6_ (Figure 5a) were determined using the seven ordered spin states of Supplementary Figures S4 and S5, respectively. In the energy-mapping analysis, we determined the relative energies of the ordered spin states by DFT + U calculations (Supplementary Tables S2–S4), expressed the energies of these states in terms of the spin exchanges (Supplementary Tables S5–S7), and finally mapped the relative energies of the DFT + U calculations to the corresponding relative energies expressed in terms of the spin exchanges to find the values of the spin exchanges. 

The spin exchanges of A_2_Ni_2_TeO_6_ (A = K, Li) and Li_3_Ni_2_SbO_6_ obtained from the energy-mapping analysis are summarized in Table 5 and Table 6, from which we note the following trends: (1) the first NN spin exchanges are all strongly FM, (2) the second NN spin exchanges are all negligible, and (3) the third NN spin exchanges are all strongly AFM. The magnetic order that accommodates these three factors is a zigzag order, as depicted in Figure 1b, which is what has been experimentally observed for A_2_Ni_2_TeO_6_ (A = Li, K) and A_3_Ni_2_SbO_6_ (A = Li, Na) [13,14]. To understand the occurrence of a short-range magnetic order in these materials, one might consider every FM chain of their zigzag-ordered honeycomb lattices as a pseudo-spin unit. Then, each zigzag-ordered honeycomb lattice is equivalent to an AFM uniform chain, so the short-range order of the zigzag-ordered honeycomb lattice becomes equivalent to that of an AFM uniform chain. Namely, the short-range order in the zigzag-ordered honeycomb lattices of A_2_Ni_2_TeO_6_ (A = Li, K) and A_3_Ni_2_SbO_6_ (A = Li, Na) is associated with short-range ordering of the FM chains along the direction perpendicular to the chains.

**Figure 6:**
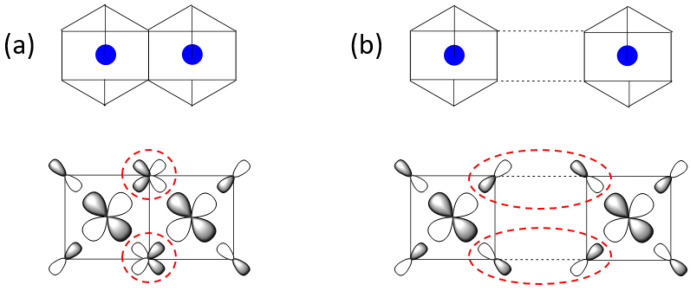
Arrangement of two x^2^ − y^2^ magnetic orbitals (**a**) in the first NN exchange path and (**b**) in the third NN exchange path in a honeycomb lattice of magnetic ions. In (**a**,**b**), the two magnetic orbitals interact through their p-orbital tails in the circled regions. The NiO_6_ octahedra are presented to emphasize the square planes containing the x^2^ − y^2^ magnetic orbitals.

It is of interest to examine why the zigzag order arises in the honeycomb lattices of A_2_Ni_2_TeO_6_ (A = Li, K) and A_3_Ni_2_SbO_6_ (A = Li, Na). The magnetic orbitals of the Ni^2+^ ion in a NiO_6_ octahedron are the e_g_ states, namely, the x^2^ − y^2^ and 3z^2^ − r^2^ states, which are combined out-of-phase with the 2p orbitals of the surrounding oxygen ligands. Of the two, the x^2^ − y^2^ state can have a substantial interaction in the first and third NN exchange paths, as depicted in Figure 6a,b, respectively, because the NiO_4_ square planes containing these orbitals can be coplanar. The first NN exchange consists of two Ni-O-Ni paths, in which the two x^2^ − y^2^ states have their p-orbitals orthogonally arranged at the shared oxygen atoms (Figure 6a). Thus, between the two magnetic orbitals, the overlap integral is zero while the overlap density is nonzero. As a result, the first NN exchange becomes FM [15,20,21,45]. The third NN exchange consists of two Ni-O⋅⋅⋅O-Ni paths, in which the p-orbital tails of the two x^2^ − y^2^ states are arranged such that the overlap integral is nonzero while the overlap density is practically zero. As a result, the third NN exchange becomes AFM. For the second NN exchange, the NiO_4_ square planes containing the x^2^ − y^2^ states cannot be coplanar (see Supplementary Figure S6). Thus, neither the overlap integral nor the overlap density between the two x^2^ − y^2^ states can be substantial, so the second NN exchange is weak.

Finally, we examined the effective interlayer spin exchanges in A_2_Ni_2_TeO_6_ (A = K, Li) and Li_3_Ni_2_SbO_6_. What matters in a long-range magnetic ordering in these systems at low temperature is whether the zigzag-ordered honeycomb layers become ferromagnetically or antiferromagnetically ordered (Supplementary Figures S7–S9). Results of these calculations are summarized in Table 7, which predicts that the honeycomb layers should be ferromagnetically coupled. This is in agreement with the experiment for Li_3_Ni_2_SbO_6_ [13]. The preference for the FM interlayer coupling is much stronger for Li_2_Ni_2_TeO_6_ than for K_2_Ni_2_TeO_6_. This reflects that the interlayer distance is shorter for Li_2_Ni_2_TeO_6_, which strengthens the interlayer interaction. This result is consistent with the observation that the long-range ordering temperature, *T*_N_, which involves the ordering between the zigzag-ordered honeycomb layers, is greater for Li_2_Ni_2_TeO_6_ than for K_2_Ni_2_TeO_6_ (~24.4 vs. ~22.8 K).

## 4. Concluding Remarks

The static magnetic susceptibility along with specific heat data showed the onset of antiferromagnetic order at *T_N_* ≈ 22.8 and 24.4 K for K_2_Ni_2_TeO_6_ and Li_2_Ni_2_TeO_6_, respectively, which is preceded by a short-range order. The high-temperature magnetic susceptibility data exhibited Curie–Weiss behavior, with Weiss temperatures Θ of approximately −13 and −20 K for K_2_Ni_2_TeO_6_ and Li_2_Ni_2_TeO_6_, respectively. The effective magnetic moment was estimated to be about 4.46 *μ*_B_ per formula unit and agrees with the theoretical value expected for Ni^2+^ (*S* = 1) ions. If we were to describe the high-temperature magnetic portion of the susceptibilities of A_2_Ni_2_TeO_6_ (A = K, Li), they can be described by a honeycomb with spin exchange *J* ≈ −8 ± 1 K. ESR spectra in the paramagnetic phase showed a single Lorentzian-shaped line, which was attributed to Ni^2+^ ions at octahedral sites, which were characterized by the isotropic effective *g*-factor 2.20 ± 0.01. In addition, our ESR data indicated an extended region of short-range order correlations, typical of low-dimensional or frustrated magnets. The intralayer spin exchanges evaluated for A_2_Ni_2_TeO_6_ (A = K, Li) and Li_3_Ni_2_SbO_6_ showed that the honeycomb layers of these magnets adopted a zigzag order, largely because the third nearest-neighbor spin exchanges are strongly antiferromagnetic and because the first nearest-neighbor spin exchanges are strongly ferromagnetic. This finding arises largely from the fact that the spin exchanges between adjacent Ni^2+^ ions are governed largely by their x^2^ − y^2^ magnetic orbitals. Adjacent zigzag-ordered honeycomb layers prefer to be ferromagnetically than antiferromagnetically coupled. The short-range order of the zigzag-ordered honeycomb lattice is equivalent to that of an antiferromagnetic uniform chain, and arises from the short-range ordering of the ferromagnetic chains along the direction perpendicular to the zigzag chains.

## Figures and Tables

**Table 1:** Lattice parameters of Li_2_Ni_2_TeO_6_ (*Cmca*) from different sources.

Source	a, Å	b, Å	c, Å	V, Å^3^
[16]	8.9667 (18)	5.1574 (14)	10.1878 (26)	471.1
[17]	8.9925 (4)	5.1469 (2)	10.1691 (5)	470.7
This work	8.9945 (19)	5.1488 (12)	10.1628 (18)	470.6

**Table 2:** Average lengths of the Li-O, Ni-O, and Te-O bonds and oxidation states of Li, Ni, Te, and O of Li_2_Ni_2_TeO_6_ expected.

(a) Average bond lengths			
Bonds	Sum of Ionic Radii [24]	X-ray Diffraction [17]	Optimized (This Work)
Li-O	1.98	2.20	2.08
Ni-O	2.07	2.13	2.07
Te-O	1.94	1.87	1.97
(b) Oxidation states from bond valence sum analysis [25]			
	Expected ^a^	X-ray Diffraction [17]	Optimized (This Work)
Li	+1	+0.68	+0.82
Ni	+2	+2.13	+1.99
Te	+6	+6.98	+5.29
O1 (16g)	−2	−2.15	−1.87
O2 (8f)	−2	−1.70	−1.72

^a^ From the ionic electron counting scheme.

**Table 3:** Parameters describing the magnetic subsystems of A_2_Ni_2_TeO_6_ (A = K, Li) tellurates.

	Θ, K	*μ*_eff_, *μ*_B_/f.u.	*T*_max_, K	*T_N_*, K	*J*, K	*B*_SF_, T
Li_2_Ni_2_TeO_6_	−20 ± 1	4.47 ± 0.01	~34	24.4 ± 0.2	−8 ± 1	~4.7
K_2_Ni_2_TeO_6_	−13 ± 1	4.45 ± 0.01	~34	22.8 ± 0.2	−8 ± 1	~4.4

**Table 4:** The spin-dynamic parameters in A_2_Ni_2_TeO_6_ (A = K and Li) tellurates.

	Effective *g*-Factor	ΔB*, mT	*D*, mT/K	*β*
Li_2_Ni_2_TeO_6_	2.20 ± 0.03	217 ± 5	-	0.60 ± 0.05
K_2_Ni_2_TeO_6_	2.20 ± 0.03	290 ± 5	0.4	1 ± 0.05

**Table 5:** Experimental Ni…Ni distances (in Å) and calculated spin exchanges (in K), obtained from DFT + U computations, of Li_2_Ni_2_TeO_6_ and Li_3_Ni_2_SbO_6_.

	Li_2_Ni_2_TeO_6_			Li_3_Ni_2_SbO_6_		
	Ni…Ni	U_eff_ = 3 eV	U_eff_ = 4 eV	Ni…Ni	U_eff_ = 3 eV	U_eff_ = 4 eV
*J*_1_	2.961	39.57	31.41	2.983	19.6	16.1
*J*_1_′	2.999	31.39	25.05	2.995	41.7	33.0
*J*_2_	5.178	−1.93	−1.43	5.179	−0.14	−0.08
*J*_2_′	5.160	0.40	0.41	5.183	−2.5	−1.9
*J*_3_	6.019	−33.47	−26.59	5.985	−22.8	−17.7
*J*_3_′	5.949	−40.53	−31.45	5.980	−29.0	−22.8

**Table 6:** Experimental Ni…Ni distances (in Å) and calculated spin exchanges (in K), obtained from DFT + U computations, of K_2_Ni_2_TeO_6_.

	Ni…Ni	U_eff_ = 3 eV	U_eff_ = 4 eV
*J*_1_	3.035	26.8	21.2
*J*_2_	5.256	−0.8	−0.6
*J*_3_	6.069	−41.2	−32.2

**Table 7:** Relative energies (in K per formula unit), calculated from DFT + U computations, of the FM and AFM arrangements between the zigzag-ordered layers in A_2_Ni_2_TeO_6_ (A = K, Li) and Li_3_Ni_2_SbO_6_.

	Li_2_Ni_2_TeO_6_		Li_3_Ni_2_SbO_6_		K_2_Ni_2_TeO_6_	
U^eff^	3 eV	4 eV	3 eV	4 eV	3 eV	4 eV
FM	0	0	0	0	0	0
AFM	6.8	5.3	3.4	2.7	0.6	0.5

## Data Availability

The original data are available on the request.

## Supplementary Materials

The following supporting information can be downloaded at: https://www.mdpi.com/article/10.3390/ma15072563/s1, Section S1 on the crystal structure of A_2_Ni_2_TeO_6_, Section S2 on the intralayer spin exchanges, and Section S3 on the intralayer spin exchanges. It includes Figures S1–S10 and Tables S1–S7. References [16,22,46] are cited in the supplementary materials.
