# Supplementary material for: Magnetic Properties of A2Ni2TeO6 (A = K, Li): Zigzag Order in the Honeycomb Layers of Ni2+ Ions Induced by First and Third Nearest-Neighbor Spin Exchanges
Source: Materials (Basel). 2022 Mar 31;15(7):2563. doi: 10.3390/ma15072563 (PMC8999558; doi:10.3390/ma15072563)
Supplement: Supplementary file 1 [file materials-15-02563-s001.zip › materials-1638555-supplementary.pdf]

materials

MDPI

Supplementary Materials

Magnetic properties of $A_2Ni_2TeO_6$ (A = K, Li): Zigzag order in the honeycomb layers of Ni^{2+} ions induced by first and third nearest-neighbor spin exchanges

Tatyana Vasilchikova,[1] Alexander Vasiliev,[1,2,3,*] Maria Evstigneeva,[4] Vladimir Nalbandyan,[4] Ji Sun Lee,[5] Hyun-Joo Koo,[5,*] Myung-Hwan Whangbo[5,6]

[1]Department of Low Temperature Physics and Superconductivity, Lomonosov Moscow State University, Moscow, 119991, Russia
[2]Quantum Functional Materials Laboratory, National University of Science and Technology "MISiS", Moscow 119049, Russia
[3]Department of Theoretical Physics and Applied Mathematics, Ural Federal University, Ekaterinburg 620002, Russia
[4]Faculty of Chemistry, Southern Federal University, Rostov-on-Don 344090, Russia
[5]Department of Chemistry and Research Institute for Basic Sciences, Kyung Hee University, Seoul 02447, Republic of Korea
[6]Department of Chemistry, North Carolina State University, Raleigh, NC 27695, USA

S1. Sample preparation and X-ray characterization

[1] $K_2Ni_2TeO_6$

Stoichiometric amounts of dried reagent-grade K_2CO_3, NiO, and TeO_2 were carefully mixed, pressed into thin pellets (to facilitate oxidation), calcined in air for 4 h at 620 °C, then heated slowly to 700 °C, and kept at this temperature for an hour and quenched. The pellets were ground and pressed again, leaving a small amount as protective powder. Pellets covered with the powder (to minimize possible losses of volatile components) were calcined in air for 3 h at 800 °C and were quenched. The hot sintered pellets, after fast removing the sacrificial powder, were immediately placed in several tightly closed containers and stored in a desiccator.

For the powder X-ray diffraction (XRD) study, a sample pellet was quickly ground, and the resulting powder was packed into a home-made sample holder sealed with a semi-cylindrical cover made of an adhesive tape [46]. In contrast to flat covers, XRD reflections from this tape were avoided and X-ray attenuation was minimized because the tape was always normal to the X-ray beam. The XRD pattern (Fig. S1) was taken with CuKα radiation using an ARL X'tra diffractometer in conventional Bragg-Brentano geometry using solid-state Si(Li) detector eliminating all undesirable radiations. The hexagonal lattice parameters and sample displacement were refined with the CELREF 3 program (J. Laugier and B. Bochu). The strongest extra peaks, marked with arrows in Fig. S1, are attributed to basal spacings of the partially hydrated phase, because their intensities grew rapidly on exposure to air, together with a corresponding decrease in the intensities of the respective reflections from the main phase. They permitted the estimation of the c lattice parameter of the hydrate as 12.74 Å. Very weak additional reflections (question marks in Fig. S1) could not be explained unambiguously.

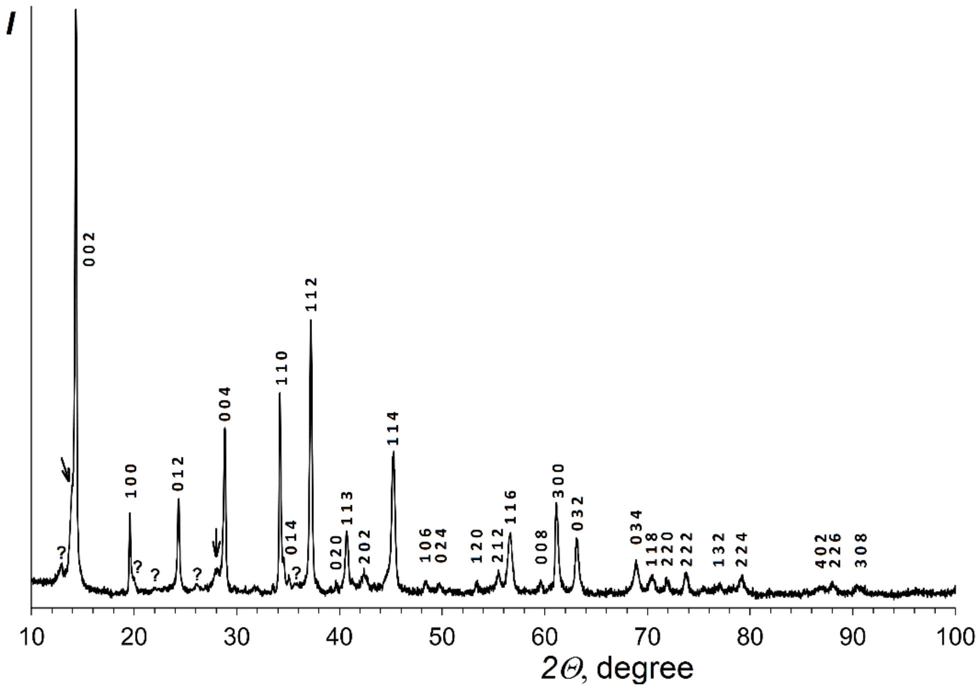

Figure S1. XRD pattern of $K_2Ni_2TeO_6$ (CuKα radiation), where reflections from the partially hydrated phase are shown by arrows, and unknown impurities, by question marks.

[2] $Li_2Ni_2TeO_6$

$Na_2Ni_2TeO_6$ was prepared first by solid-state reactions as reported previously [22], and then ion-exchanged in 10-fold excess molten $LiNO_3$ for 3 h at 300 °C. The product was washed with water and dried at 120 °C. Both Na and Li compounds are not so hygroscopic unlike their potassium counterpart, so they were handled and X-rayed without special precautions but they were still stored in a desiccator. The XRD pattern of the product is shown in Fig. S2 in comparison with the initial preparation [16].

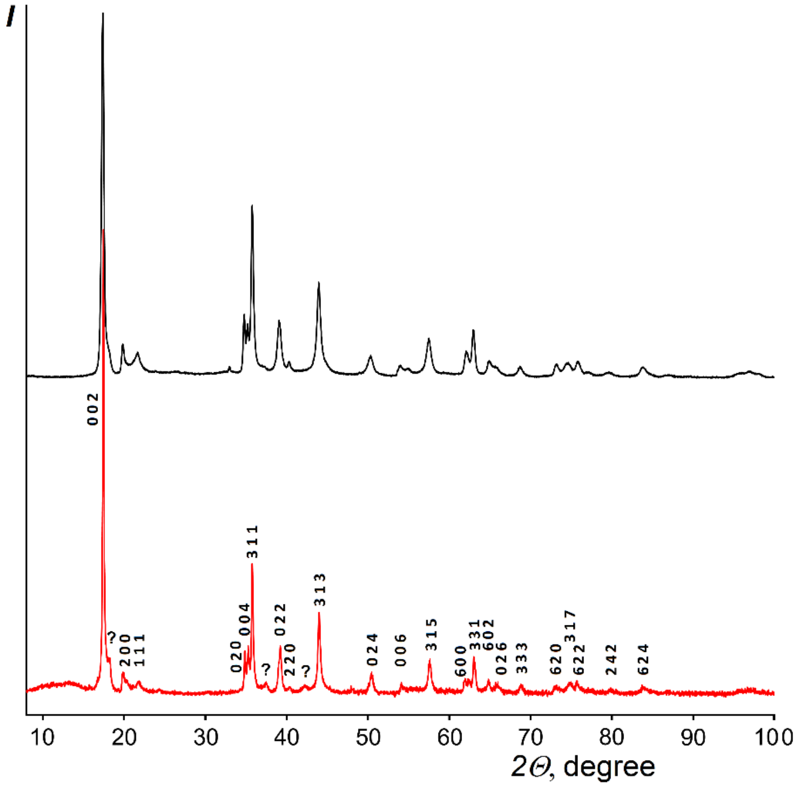

Figure S2. XRD patterns of $Li_2Ni_2TeO_6$ (CuKα radiation). The top profile in black is from our first report [16], with amorphous powder admixed to reduce the grain orientation effect (Rigaku D-Max diffractometer with graphite monochromator), and the bottom profile in red is for the sample of this work (ARL X'tra diffractometer with solid-state Si(Li) detector), which shows a considerable (*00l*) texture and where the question marks indicate unindexed reflections.

Table S1. Atomic coordinates of $Li_2Ni_2TeO_6$ (a = 8.9798 Å, b = 5.1595 Å, c = 10.2094 Å; space group *Cmca*) optimized by DFT+U calculations with U_{eff} = 4 eV.

Atom	Site	x/a	y/b	z/c
Li	8e	1/4	0.6146	1/4
Ni	8d	0.3351	0	0
Te	4a	0	0	0
O(1)	16g	0.3371	0.3505	0.1093
O(2)	8f	0	0.3157	0.1008

Copyright: © 2022 by the authors. Licensee MDPI, Basel, Switzerland. This article is an open access article distributed under the terms and conditions of the Creative Commons Attribution (CC BY) license (https://creativecommons.org/licenses/by/4.0/).

Scheme 2. Intralayer spin exchanges of the honeycomb layers.

[1] Ordered spin states employed

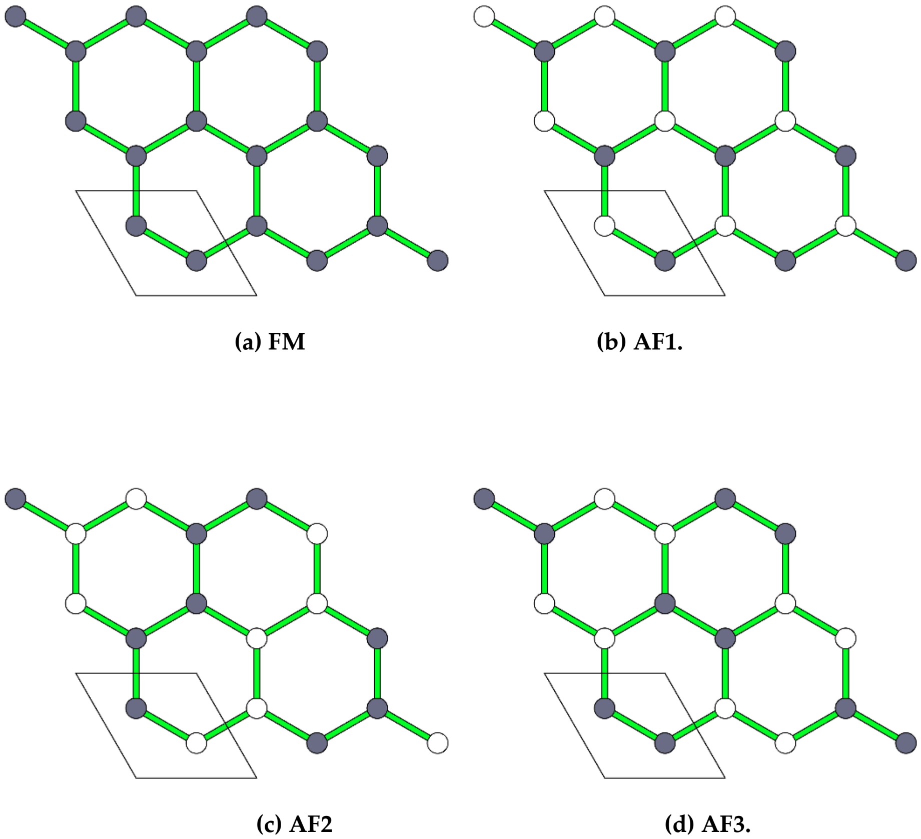

Figure S3. Ordered spin arrangements of (**a**) FM, (**b**) AF1, (**c**) AF2 and (**d**) AF3 states in $K_2Ni_2TeO_6$.

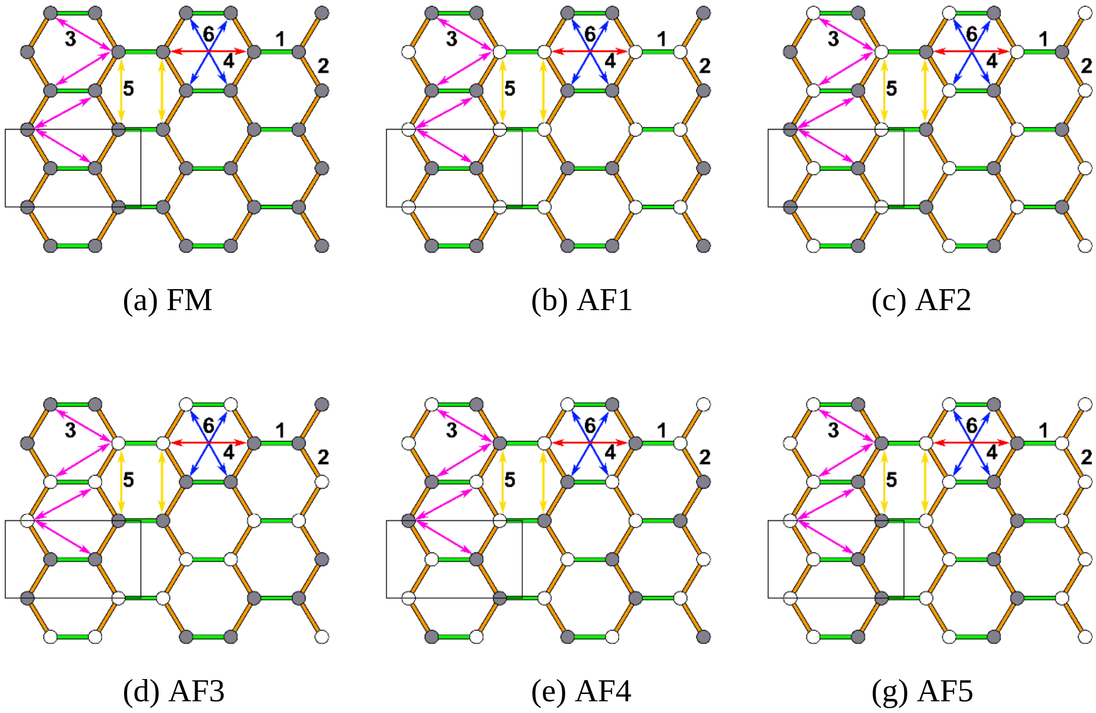

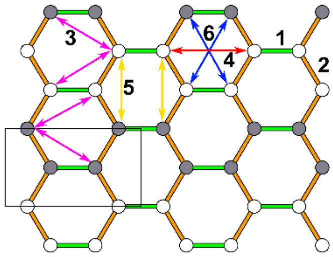

(h) AF6

Figure S4. Ordered spin arrangements of (**a**) FM, (**b**) AF1, (**c**) AF2, (**d**) AF3, (**e**) AF4, (**g**) AF5 and (**h**) AF6 states in $Li_2Ni_2TeO_6$.

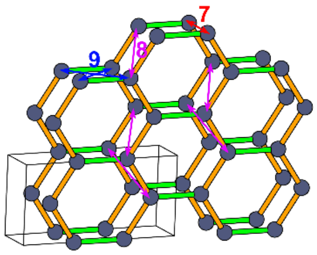

(a) FM

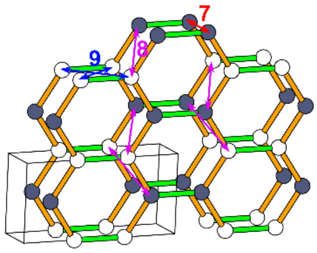

(b) AF1

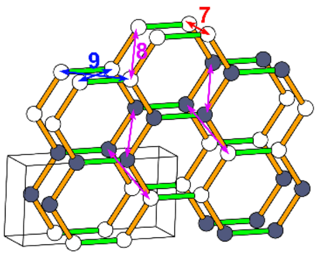

(c) AF2

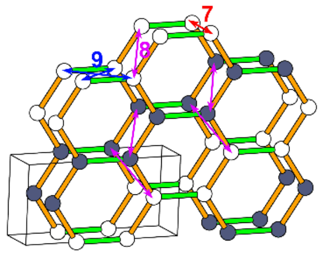

(d) AF3

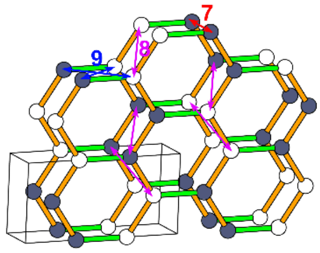

(e) AF4

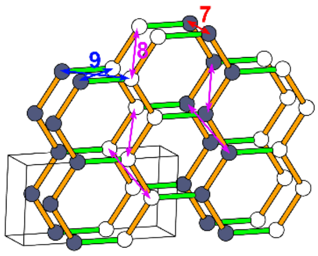

(f) AF5

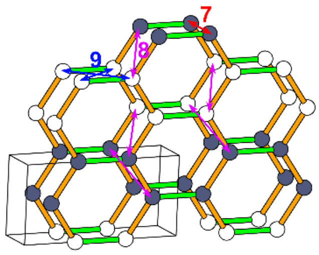

(g) AF6

Figure S5. Ordered spin arrangements in $Li_3Ni_2SbO_{12}$.

[2] Relative energies of ordered spin states

Table S2. Relative energies (meV/FU) of ordered spin states obtained from DFT+U calculations for $K_2Ni_2TeO_6$.

	**U = 3 eV**	**U = 4 eV**
E_{FM}	17.23	13.37

E_{AF1}	9.82	7.72
E_{AF2}	0	0
E_{AF3}	25.94	20.29

Table S3. Relative energies (meV/FU) of ordered spin states obtained from DFT+U calculations for the optimized crystal structure of $Li_2Ni_2TeO_6$.

	U = 3 eV	U = 4 eV
E_{FM}	14.86	11.46
E_{AF1}	24.35	19.11
E_{AF2}	12.76	10.09
E_{AF3}	0	0
E_{AF4}	20.80	16.26
E_{AF5}	0.61	0.46
E_{AF6}	5.77	4.58

Table S4. Relative energies (in meV/FU) of ordered spin states obtained from DFT+U calculations of $Li_3Ni_2SbO_{12}$.

	U = 3 eV	U = 4 eV
E_{FM}	9.42	7.25
E_{AF1}	21.56	16.93
E_{AF2}	12.34	9.79
E_{AF3}	0.69	0.54
E_{AF4}	15.33	12.11
E_{AF5}	0	0
E_{AF6}	4.63	3.58
E_{AF7}	12.42	9.86
E_{AF8}	1.11	0.85
E_{AF9}	7.69	5.92

[3] Expressions of the spin exchange energies of the ordered spin states

With the spin Hamiltonian given blow,

$$H = -\sum_{i>j} J_{ij}\vec{S}_i \cdot \vec{S}_j \qquad (S1)$$

the total spin exchange energy for a given ordered spin state can be written as

$$E = \frac{1}{N^2}\sum_i n_i J_i \qquad (S2)$$

where N refers to the number of unpaired electrons of a magnetic ion, and n_i to the number of a particular J_i path (i.e., a particular J_{ij} path in the spin Hamiltonian) that occurs in the supercell used.

Table S5. Values of n_i for the ordered spin states of $K_2Ni_2TeO_6$.

	J_1	J_2	J_3
E_{FM}	-24	-48	-24
E_{AF1}	24	-48	24
E_{AF2}	-8	16	24
E_{AF3}	8	-16	-24

Table S6. Values of n_i for the ordered spin states of $Li_2Ni_2TeO_6$.

	J_1	J_2	J_3	J_4	J_5	J_6
E_{FM}	-16	-32	-64	-16	-32	-32
E_{AF1}	-16	32	64	-16	-32	-32
E_{AF2}	16	32	-64	16	-32	32
E_{AF3}	-16	0	0	16	32	32

E_{AF4}	16	0	0	16	32	-32
E_{AF5}	16	-32	64	16	-32	32
E_{AF6}	-16			-16	32	32

Table S7. Values of n_i for the ordered spin states of $Li_3Ni_2SbO_6$.

	J_1	J_2	J_3	J_4	J_5	J_6
E_{FM}	-16	-32	-64	-16	-32	-32
E_{AF1}	-16	32	64	-16	-32	-32
E_{AF2}	16	32	-64	16	-32	32
E_{AF3}	-16			16	32	32
E_{AF4}	16			16	32	-32
E_{AF5}	16	-32	64	16	-32	32
E_{AF6}	-16			-16	32	32

S3. Interlayer spin exchanges

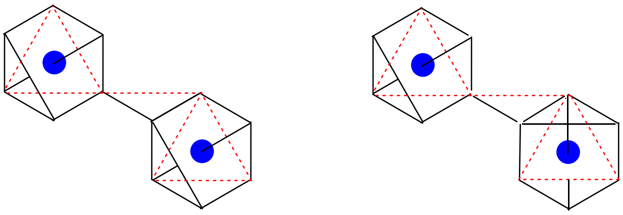

Figure S6. Two possible arrangements of the magnetic orbital planes containing the x^2-y^2 magnetic orbitals for the second NN spin exchange, where the dashed lines lying in a same plane are shown for the ease of recognizing the non-planarity of the two magnetic orbital planes.

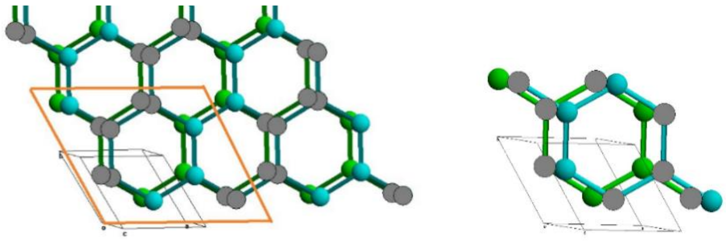

(a) FM **(b) AFM.**

Figure S7. (**a**) FM and (**b**) AFM arrangements of zigzag-ordered layers in $Li_3Ni_2SbO_{12}$, where the gray and white circles indicate up spin and down spin of Ni^{2+} ions, respectively.

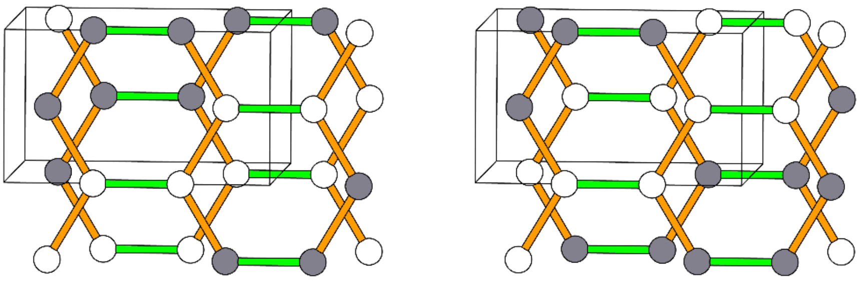

(a) FM **(b) AFM.**

Figure S8. (**a**) FM and (**b**) AFM arrangements of the zigzag-ordered layers in $Li_2Ni_2TeO_6$, where the gray and white circles indicate up spin and down spin of Ni^{2+} ions, respectively.

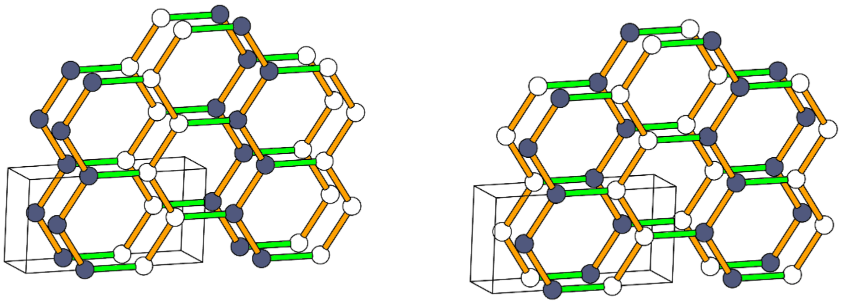

(a) FM **(b) AFM.**

Figure S9. (**a**) FM and (**b**) AFM arrangements of zigzag-ordered layers in $Li_3Ni_2SbO_{12}$, where the gray and white circles indicate up spin and down spin of Ni^{2+} ions, respectively.

S4. ESR Spectroscopy

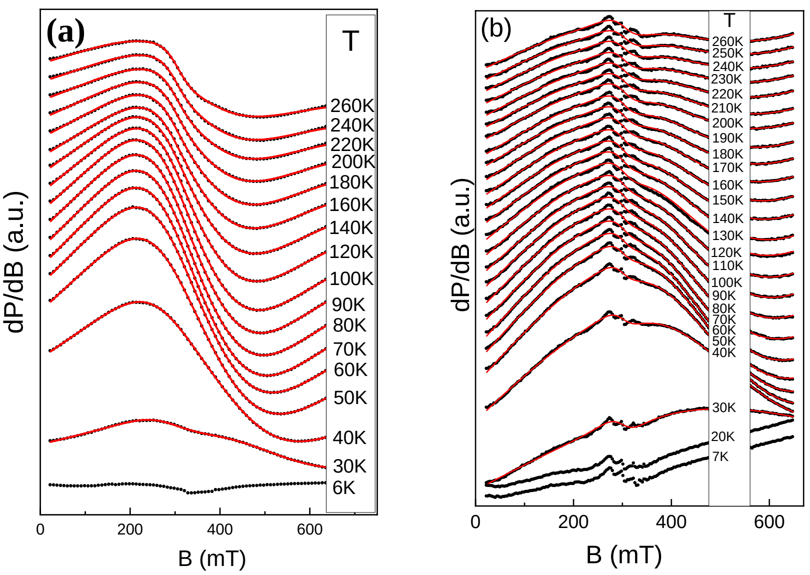

Figure S10. The temperature-dependent ESR spectra of $Li_2Ni_2TeO_6$ (**a**) and $K_2Ni_2TeO_6$ (**b**); the Lorentzian fitting profile is given by the red solid lines. Small sharp singularities in the spectra shown in (b) panel are due to extrinsic effect.

References

16. *Powder Diffraction File*; International Centre for Diffraction Data, Newtown Square, PA, USA, **2009**, entry 00-059-0445.

22. Evstigneeva, M. A.; Nalbandyan, V. B.; Petrenko, A. A.; Medvedev, B. S.; Kataev, A. A. A New Family of Fast Sodium Ion Conductors: $Na_2M_2TeO_6$ (M = Ni, Co, Zn, Mg). *Chem. Mater.* **2011**, *23*, 1174–1181.

46. Sudorgin, N. G.; Nalbandyan, V. B. Quantitative X-ray monitoring of electrode processes in sealed cells. Reduction of zirconium β-molybdate by lithium. *Soviet electrochem.* **1992**, *28*, 100–102.
